# Adherence, healthcare resource utilization and Medicaid spending associated with once-monthly paliperidone palmitate versus oral atypical antipsychotic treatment among adults recently diagnosed with schizophrenia

**DOI:** 10.1186/s12888-017-1358-3

**Published:** 2017-06-02

**Authors:** Dominic Pilon, Erik Muser, Patrick Lefebvre, Rhiannon Kamstra, Bruno Emond, Kruti Joshi

**Affiliations:** 1 Groupe d’analyse, Ltée, 1000 De La Gauchetière West, Suite 1200, Montréal, QC H3B 4W5 Canada; 2 Janssen Scientific Affairs, LLC, Titusville, NJ USA

## Abstract

**Background:**

Once-monthly paliperidone palmitate (PP1M) is a long-acting injectable antipsychotic that may increase adherence rates, reduce hospitalizations, and lower medical costs compared to oral atypical antipsychotics (OAAs) among schizophrenia patients. However, the impact of PP1M in recently diagnosed patients remains unknown. The present study compared adherence, healthcare resource utilization and Medicaid spending between schizophrenia patients initiating PP1M versus OAA, among patients recently diagnosed (defined using ages 18–25 years as a proxy) and among the overall population.

**Methods:**

Medicaid data from five states (09/2008–03/2015) were used to identify adults with schizophrenia initiated on PP1M or OAAs (index date) on or after 09/2009. Outcomes were compared between PP1M and OAA groups following inverse probability of treatment weighting (IPTW). Univariate linear and Poisson regression models with nonparametric bootstrap procedures were used to compare the 12-month healthcare resource utilization and costs using rate ratios (RRs) and mean monthly cost differences (MMCDs), respectively.

**Results:**

Overall, patients initiated on PP1M (*N* = 2053) were younger (mean age: 41 vs. 44 years) and had more baseline antipsychotic use (88% vs. 62%) compared to OAA patients (*N* = 22,247). IPTW resulted in balanced baseline characteristics. Among recently diagnosed patients, PP1M was associated with better adherence (PDC ≥ 80%: 29% vs. 21%, *P* < 0.001) on the index medication as well as less use of other psychiatric medications, compared to OAAs. Adherence findings were similar for the overall cohort. Among recently diagnosed patients, lower medical costs associated with PP1M (MMCD = $-466; *P* = 0.028) outweighed the higher pharmacy costs (MMCD = $322; *P* < 0.001) resulting in similar total healthcare costs across groups (MMCD = $-144; *P* = 0.553). Overall, findings were similar but there was a trend toward a lower magnitude of medical cost savings (MMCD = $-286; *P* < 0.001). Reductions in medical costs were mainly driven by reductions in inpatient days (recently diagnosed RR = 0.85, *P* = 0.353; overall RR = 0.84, *P* = 0.004) and in home care visits (recently diagnosed RR = 0.43, *P* = 0.008; overall RR = 0.78, *P* = 0.048).

**Conclusions:**

PP1M patients demonstrated significantly lower medical costs offsetting higher pharmacy costs relative to OAA patients. Recently diagnosed patients using PP1M may have greater medical cost savings relative to OAAs than that observed in the overall population, highlighting the potential economic impact of PP1M in adults recently diagnosed with schizophrenia.

## Background

Schizophrenia is a debilitating chronic mental illness which is one of the top 20 leading causes of disability worldwide and is associated with a high cost burden [1, 2]. The overall cost of schizophrenia in the United States (US) in 2013 was estimated at $155.7 billion, with nearly one-quarter of this cost incurred as direct healthcare costs [2]. The large burden of schizophrenia can be attributed in part to the early onset of the disease, typically in the early-to-mid 20s for men and in the late 20s for women; which then frequently persists as a chronic, lifelong illness [3]. Therefore, patients with recently diagnosed schizophrenia, including younger patients for whom disease onset is very likely to be recent, represent an important population for developing and targeting interventions.

Suboptimal disease management and multiple relapses have been associated with a poor functional prognosis [4] and reduced responsiveness to antipsychotic (AP) therapy [4, 5], and can beget a cycle of economic, social, and legal challenges that pose further barriers to effective intervention [6]. Therefore, treating patients early in the course of disease, when they may be most treatable, is of high priority to minimize the risk of serious long-term consequences [7]. While antipsychotic (AP) therapy is an effective tool in managing the symptoms of schizophrenia and preventing relapse, non-adherence to therapy is common [6]. Moreover, patients early in the course of disease may be particularly at risk for non-adherence, due in part to lack of insight into their own illness [8, 9].

Although the determinants of non-adherence are numerous and complex, medication-related factors such as the choice of AP therapy and dosing frequency are among its important drivers [6, 10]. For example, long-acting injectable therapies (LAIs) can be administered less frequently (once every 1 to 12 weeks) than daily oral APs and are typically healthcare provider-administered, meaning that once the injection is received, no further action is required by the patient for the duration of that dose. Current evidence suggests that for patients with schizophrenia in general, using second-generation LAIs may be associated with lower rates of disease relapse and reduced morbidity when compared to oral atypical APs (OAA), likely through increasing medication adherence [11–14]. Once-monthly paliperidone palmitate (PP1M) is one such LAI that was approved in 2009 by the FDA for the treatment of schizophrenia in adults and in 2014 by the FDA for the treatment of schizoaffective disorder [15].

It has been previously demonstrated that recently diagnosed adults with schizophrenia can tolerate and be safely and effectively treated with LAIs including PP1M [16–18]. Notably, a recent post-hoc analysis of trial data has suggested that the magnitude of the impact of PP1M versus oral APs on treatment failure may be greater among patients with recent-onset illness than among chronic patients [18]. Importantly, this was an underpowered secondary analysis; therefore, there is a need for real-world evidence addressing whether LAIs could increase rates of adherence and influence subsequent economic outcomes in patients with recently diagnosed schizophrenia. This study aims to evaluate adherence, healthcare resource utilization, and Medicaid spending in patients with schizophrenia treated with PP1M as compared to those being treated with OAAs among a five-state Medicaid population, among patients aged 18 to 25 years as a proxy for being recently diagnosed with schizophrenia and among the overall cohort of patients with schizophrenia.

## Methods

### Data source

This study was conducted using Medicaid healthcare claims databases from New Jersey, Iowa, Missouri, Mississippi, and Kansas. Data were available from September 1, 2008 through March 31, 2015 for all states except for New Jersey, which had available data until March 31, 2014. The available data included patient eligibility records (e.g., age, gender, race, enrollment start/end dates), medical claims (e.g., type of service, date of service, International Classification of Diseases, Ninth Revision, Clinical Modification [ICD-9-CM] diagnoses, Current Procedural Terminology [CPT] procedure codes, and Healthcare Common Procedure Coding System [HCPCS] codes), and prescription drug claims (e.g., days of medication supplied, date of dispensing, and National Drug Codes [NDC]). All available cost data reflect the Medicaid payers’ perspective prior to any discounts or rebates paid by manufacturers. All data were de-identified and in compliance with the Health Insurance Portability and Accountability Act (HIPAA).

### Study design and patient selection

A retrospective longitudinal cohort study was conducted using adult Medicaid beneficiaries with schizophrenia to compare treatment patterns, healthcare resource utilization, and Medicaid spending in patients who were initiated on PP1M versus OAAs. The treatments of interest in this study included PP1M and nine FDA-approved OAAs (i.e., aripiprazole, asenapine maleate, iloperidone, lurasidone, olanzapine, paliperidone, quetiapine fumarate, risperidone, and ziprasidone).

To be included in the study, patients were required to meet the following criteria: (a) have at least two pharmacy/medical claims for PP1M or at least two pharmacy claims for the same OAA agent within 90 days starting on or after September 1, 2009 with no claims in the previous 12 months (baseline period; the date of the first claim was defined as the index date), (b) have at least two diagnoses for schizophrenia (ICD-9-CM codes: 295.xx) during the study period, (c) be at least 18 years of age on the index date, (d) have at least 12 months of continuous Medicaid enrollment prior to the index date, and (e) have at least 12 months of post-index continuous Medicaid enrollment. Outcomes were evaluated during a fixed 12-month observation period including and following the index date. Baseline demographics and clinical characteristics were evaluated during the 12-month period prior to the index date.

Treatment cohorts were defined by the agent initiated on the index date. Agents were identified using the generic product identifier (GPI) code and/or the HCPCS code. All outcomes were evaluated both for PP1M and OAA patients aged 18–25 years at the time of diagnosis, using age as a proxy for recent schizophrenia diagnosis, and for the overall cohort (aged ≥18 years).

### Study outcomes

Outcomes included treatment patterns, all-cause healthcare resource utilization, and healthcare costs, measured over the 12-month observation period for each patient and compared between PP1M and OAA treatment groups.

Treatment patterns, evaluated during the observation period, were described. They included the duration of continuous exposure to the index agent (spanning from the index date to the end of the days of supply of the first fill for which the next fill of the index treatment, if any, is >90 days later), the number of dispensings of the index agent, psychiatric medication use (other than the index agent) by type, the presence of AP polypharmacy (i.e., overlapping coverage of ≥2 unique AP agents for ≥60 consecutive days with no more than a 7-day gap), and the presence of psychiatric polypharmacy (i.e., overlapping coverage of ≥1 AP and ≥1 anxiolytic, antidepressant, or mood stabilizer for ≥60 consecutive days with no more than a 7-day gap). Adherence (using the proportion of days covered [PDC] with ≥80% to define adherent patients) and persistence (using no gap ≥30, 60, or 90 days) were also evaluated at 12 months post-index date for any AP as well as for the index agent. PDC was defined as the number of non-overlapping days of supply divided by the number of days in the observation period (365 days).

For healthcare resource utilization, the frequency of visits/services was evaluated by type, in addition to the length of stay which was evaluated for inpatient visits, long-term care admissions, and mental health institute admissions (> 1 day). Healthcare costs were evaluated for medical costs by type of service (i.e., inpatient visits, outpatient visits, emergency room visits, long-term care admissions, mental health institute admissions [> 1 day], 1-day mental health institute admissions, home health care services, and other medical ancillary services) as well as for total pharmacy costs. Costs were based on the Medicaid payers’ perspectives and reflect amounts paid by state Medicaid programs prior to discounts or rebates. As a sensitivity analysis, a 23.1% discount (the mandatory minimum discount for branded pharmaceutical products in Medicaid) [19] was applied to all pharmacy claims for branded pharmaceuticals for the estimation of total healthcare costs and total pharmacy costs.

### Statistical analysis

#### Inverse probability of treatment weighting

In an observational study setting, many factors may be associated with whether a patient receives one treatment versus another, likely resulting in important differences between the characteristics of the comparison groups (i.e., PP1M-treated vs. OAA-treated patients) which could confound the observed effect of the treatment on the outcomes. To minimize the effect of potential confounding factors without reducing the size of the study population, inverse probability of treatment (IPT) weighting was used to adjust for differences in baseline characteristics (i.e., evaluated during the 12-month period prior to the index date) and compare outcomes between PP1M and OAA treatment groups. Weights were calculated based on propensity scores (PS): 1/PS for the PP1M cohort and 1/(1-PS) for the OAA cohort and normalized by dividing each weight by the mean. PS were estimated using multivariate logistic regression adjusted for the following baseline demographics and clinical characteristics: age, sex, race, state, region (i.e., urban, suburban, or rural), insurance eligibility (i.e., presence of capitated and/or dual coverage), year and quarter of index date, Quan-Charlson Comorbidity Index (CCI) [20, 21], number of unique mental health diagnoses, number of unique psychiatric agents received, use of AP medications by type (i.e., typical vs. atypical and long-acting injectable vs. oral), level of AP adherence (i.e., PDC < 80%), presence of AP polypharmacy, other psychiatric medication use (i.e., anxiolytics, antidepressants, or mood stabilizers), presence of specific comorbidities (i.e., cardiovascular disease, diabetes, obesity, drug abuse, hepatitis C, HIV/AIDS), and the numbers of baseline mental health institute admissions and 1-day mental health institute admissions. PS for the recently diagnosed cohort of patients were estimated separately within this population.

Unweighted and IPT-weighted descriptive statistics were generated to summarize the baseline demographics and clinical characteristics of the study population. Percentages were used to summarize categorical variables, while means, medians, and standard deviations were used for continuous variables. Standardized differences were used to compare baseline demographics and clinical characteristics between weighted cohorts and assess the quality of the IPT-weighting, with the goal being to achieve clinically and statistically well-balanced cohorts according to an accepted threshold of ≤10% [22–24].

#### Outcome comparisons

Weighted descriptive statistics were generated to summarize the treatment patterns. Percentages were used to summarize categorical variables, while means, medians, and standard deviations were used for continuous variables. Treatment pattern-related variables were compared between cohorts using the Pearson chi-square test (categorical variables) or Student’s t-test (continuous variables) after weighting.

The 12-month cost outcomes were compared between cohorts using mean monthly cost differences (MMCD) which were estimated using weighted ordinary least squares (OLS) regression models including a binary indicator for the treatment group. OLS was used given that it provides estimates that are straightforward to interpret and correspond to the crude difference in means when performed without adjustment. All costs were standardized using the medical care component of the Consumer Price Index and reported as 2015 US dollars. Rates (number of events/days per person-year of observation) of healthcare resource utilization were compared between cohorts with the use of rate ratios (RRs) which were estimated using weighted Poisson regression models which included only a binary indicator for treatment group. Because cost and resource utilization data are positive values that follow a non-normal distribution and also often have zero values, *P*-value and 95% confidence intervals (CIs) for cost and resource utilization outcomes were estimated using a non-parametric bootstrap procedure (499 bootstrap replications).

No adjustments were made for multiple comparisons. All analyses were performed using SAS software, Version 9.3 of the SAS System for Windows, SAS Institute Inc., Cary, NC, USA.

## Results

### Demographic and clinical characteristics

**Fig. 1:**
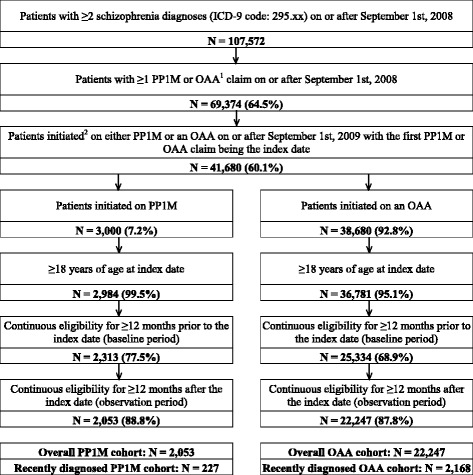
Study population flowchart for Medicaid patients with schizophrenia initiated on PP1M or OAA. PP1M = once-monthly paliperidone palmitate; OAA = oral atypical antipsychotics. Data source: Medicaid database: Iowa, Kansas, Mississippi, Missouri, and New Jersey. ^1^OAA agents include aripiprazole, asenapine, iloperidone, lurasidone, olanzapine, paliperidone, quetiapine, risperidone, and ziprasidone. ^2^Initiation of an antipsychotic agent is defined as having ≥2 claims for the same agent within 90 days and no claim of the same agent during the 12 months before the first claim

A total of 24,300 patients met the inclusion and exclusion criteria (Fig. 1). Among recently diagnosed patients, 227 and 2168 were treated with PP1M and OAA, respectively (IPT-weighted population: *N* = 1107 PP1M and *N* = 1288 OAA)**.** The overall study population consisted of 2053 patients initiated on PP1M and 22,247 initiated on OAA resulting in a corresponding IPT-weighted cohort of 11,612 PP1M patients and 12,688 OAA patients**.** Tables 1 and 2 present the baseline characteristics for unweighted and IPT-weighted cohorts for the recently diagnosed and the overall cohort, respectively.

**Table 1:** Demographic and clinical characteristics evaluated during the 12-month baseline period in unweighted and IPT-weighted recently diagnosed cohorts

	Recently diagnosed cohort					
	Unweighted			IPT-weighted		
	PP1M (*N* = 227)	OAA (*N* = 2168)	*Std. Diff.* ^a^	PP1M (*N* = 1107)	OAA (*N* = 1288)	*Std. Diff.* ^a^
Age at index date (years), mean ± SD [median]	22.3 ± 1.9 [22.5]	21.6 ± 2.0 [21.7]	31.7%	21.7 ± 2.0 [21.8]	21.7 ± 2.0 [21.8]	0.8%
Female, %	24.7%	41.1%	35.4%	27.9%	39.2%	24.1%
Race, %						
White	43.2%	52.9%	19.5%	49.4%	51.9%	5.0%
Black	44.1%	34.4%	19.9%	36.6%	35.3%	2.7%
Hispanic	0.0%	0.5%	9.6%	0.0%	0.5%	9.5%
Other	5.7%	7.2%	6.0%	5.6%	7.2%	6.2%
Unknown	7.0%	5.1%	8.1%	8.4%	5.2%	12.6%
State, %						
Iowa	6.6%	12.0%	18.6%	12.8%	11.4%	4.3%
Kansas	13.7%	8.5%	16.4%	10.1%	9.2%	2.9%
Mississippi	13.2%	12.8%	1.2%	14.2%	13.0%	3.5%
Missouri	48.5%	43.2%	10.6%	41.1%	43.5%	4.9%
New Jersey	18.1%	23.5%	13.4%	21.8%	22.9%	2.5%
Region characteristics, %						
Urban	54.6%	52.3%	4.7%	47.0%	52.1%	10.3%
Suburban	27.8%	27.1%	1.4%	33.0%	27.2%	12.5%
Rural	17.6%	20.6%	7.6%	20.0%	20.6%	1.5%
Insurance eligibility, %						
Capitated or dual coverage	63.0%	53.2%	20.0%	56.6%	53.9%	5.3%
Capitated	48.9%	44.1%	9.5%	47.7%	44.9%	5.5%
Dual coverage	22.9%	15.5%	19.0%	13.4%	15.8%	6.9%
Year of index date, %						
2009	13.7%	17.7%	11.0%	11.7%	17.1%	15.3%
2010	43.2%	38.1%	10.3%	41.0%	38.6%	5.0%
2011	19.4%	21.4%	5.1%	16.7%	21.1%	11.1%
2012	13.2%	14.2%	2.7%	19.1%	14.3%	13.0%
2013	9.3%	7.1%	7.7%	9.7%	7.6%	7.8%
2014	1.3%	1.5%	1.3%	1.7%	1.5%	1.9%
Quan-CCI, mean ± SD [median]	0.3 ± 0.6 [0.0]	0.4 ± 0.8 [0.0]	11.5%	0.4 ± 0.7 [0.0]	0.4 ± 0.8 [0.0]	6.3%
Number of unique mental health diagnoses, mean ± SD [median]	8.1 ± 7.1 [6.0]	8.5 ± 8.1 [6.5]	5.3%	8.9 ± 7.8 [7.0]	8.4 ± 8.0 [7.0]	5.4%
Number of unique AP agents received, mean ± SD [median]	1.8 ± 1.2 [2.0]	1.0 ± 1.1 [1.0]	63.8%	1.2 ± 1.2 [1.0]	1.1 ± 1.1 [1.0]	13.9%
AP use, %	88.1%	59.9%	67.9%	66.6%	62.8%	8.1%
Typical oral and short-term injectable	24.7%	21.0%	8.7%	23.5%	21.5%	5.0%
Atypical oral and short-term injectable	68.3%	50.3%	37.2%	55.6%	52.1%	7.0%
Typical LAI	12.8%	4.2%	30.9%	7.3%	5.2%	8.6%
Atypical LAI	35.7%	5.3%	81.4%	10.5%	8.5%	6.7%
Proportion of days covered (PDC) by any AP agent, %						
PDC < 0.8^b^	69.5%	69.5%	0.0%	70.7%	69.5%	2.4%
PDC ≥ 0.8^b^	30.5%	30.5%	0.0%	29.3%	30.5%	2.4%
AP polypharmacy present^c^, %	19.8%	8.8%	32.0%	11.8%	9.9%	6.1%
Other psychiatric medication use, %	74.4%	69.3%	11.4%	68.9%	69.9%	2.0%
Antidepressants	47.6%	50.8%	6.4%	49.3%	50.6%	2.5%
Anxiolytics	35.7%	35.4%	0.6%	34.2%	35.5%	2.7%
Mood stabilizers	48.5%	42.1%	12.9%	42.6%	42.8%	0.5%
Number of unique psychiatric agents received^d^, mean ± SD [median]	3.6 ± 2.4 [3.0]	2.9 ± 2.7 [2.0]	24.3%	3.0 ± 2.6 [3.0]	3.0 ± 2.7 [3.0]	0.7%
Psychiatric polypharmacy present^e^, %	42.3%	32.2%	21.0%	31.3%	33.5%	4.8%
Specific comorbidities present^f^, %						
Cardiovascular disease^g^	5.3%	6.5%	5.2%	3.2%	6.3%	14.4%
Diabetes	9.3%	6.8%	8.9%	9.4%	7.4%	7.3%
Obesity	10.1%	10.9%	2.5%	18.0%	11.0%	19.8%
Drug abuse	25.1%	20.2%	11.7%	27.4%	20.7%	15.5%
Hepatitis C^g^	0.0%	0.8%	12.6%	0.0%	0.8%	12.7%
HIV/AIDS	0.0%	0.5%	9.6%	0.0%	0.4%	9.5%
Baseline monthly healthcare costs (2015 $US), mean ± SD [median]						
Total healthcare costs	$2308 ± $3427 [$1408]	$2493 ± $5096 [$1152]	4.3%	$2422 ± $4173 [$1381]	$2526 ± $5012 [$1190]	2.3%
Total pharmacy costs	$507 ± $644 [$249]	$325 ± $747 [$86]	26.1%	$359 ± $561 [$66]	$340 ± $735 [$97]	2.9%
Total medical costs	$1802 ± $3215 [$920]	$2169 ± $4941 [$845]	8.8%	$2063 ± $3923 [$933]	$2186 ± $4858 [$885]	2.8%
Baseline healthcare resource utilization, % with ≥1 visit/service						
Outpatient visits	89.9%	89.3%	1.7%	88.3%	89.5%	3.7%
Number of visits per month, mean ± SD [median]	0.92 ± 1.05 [0.50]	1.03 ± 1.61 [0.58]	8.0%	0.90 ± 1.10 [0.50]	1.03 ± 1.62 [0.58]	9.1%
Emergency room visits	54.2%	61.2%	14.2%	56.5%	60.8%	8.7%
Number of visits per month, mean ± SD [median]	0.21 ± 0.47 [0.08]	0.21 ± 0.49 [0.08]	0.6%	0.27 ± 0.55 [0.08]	0.21 ± 0.48 [0.08]	12.4%
Inpatient visits	52.4%	54.6%	4.4%	58.2%	55.1%	6.4%
Number of visits per month, mean ± SD [median]	0.15 ± 0.39 [0.08]	0.12 ± 0.23 [0.08]	6.7%	0.14 ± 0.33 [0.08]	0.13 ± 0.24 [0.08]	6.2%
Long-term care admissions	2.2%	5.5%	17.3%	2.7%	5.7%	15.1%
Number of admissions per month, mean ± SD [median]	0.00 ± 0.02 [0.00]	0.02 ± 0.13 [0.00]	17.9%	0.00 ± 0.02 [0.00]	0.02 ± 0.12 [0.00]	18.3%
Mental health institute admissions	63.0%	36.8%	54.3%	49.7%	39.0%	21.8%
Number of admissions per month, mean ± SD [median]	0.56 ± 0.95 [0.17]	0.27 ± 0.71 [0.00]	34.0%	0.39 ± 0.75 [0.00]	0.31 ± 0.76 [0.00]	10.6%
One-day mental health institute admissions	70.9%	46.8%	50.5%	56.2%	49.0%	14.4%
Number of admissions per month, mean ± SD [median]	1.18 ± 1.22 [0.92]	0.51 ± 0.91 [0.00]	62.0%	0.73 ± 1.04 [0.17]	0.58 ± 1.00 [0.00]	14.4%
Home care	22.9%	29.0%	14.0%	19.9%	29.1%	21.5%
Number of services per month, mean ± SD [median]	0.55 ± 2.38 [0.00]	0.89 ± 3.74 [0.00]	10.7%	0.34 ± 1.73 [0.00]	0.88 ± 3.70 [0.00]	18.7%
Other services	30.8%	33.9%	6.5%	34.9%	33.6%	2.6%
Number of services per month, mean ± SD [median]	0.08 ± 0.23 [0.00]	0.09 ± 0.29 [0.00]	3.2%	0.07 ± 0.16 [0.00]	0.09 ± 0.29 [0.00]	9.5%

*AP* antipsychotics, *CCI* Charlson comorbidity index, *IPT* inverse probability of treatment, *LAI* long-acting injectable therapy, *OAA* oral atypical antipsychotics, *PDC* proportion of days covered, *PP1M* once-monthly paliperidone palmitate

^a^Baseline characteristics with a standardized difference <10% were considered well balanced. For continuous variables, the standardized difference is calculated by dividing the absolute difference in means of the PP1M and the OAA cohorts by the pooled standard deviation of both groups. The pooled standard deviation is the square root of the average of the squared standard deviations. For categorical variables with 2 levels, the standardized difference is calculated using the following equation where P is the respective proportion of participants in each group: (P_PP1M_-P_OAA_)/√[p(1-p)], where p = (P_PP1M_ + P_OAA_)/2

^b^The denominator for the proportion of patients with a PDC <0.8 or a PDC ≥0.8 is the number of patients with a PDC >0

^c^AP polypharmacy is defined as having overlapping coverage of ≥2 unique AP agents for at least 60 consecutive days with no gaps larger than 7 days

^d^Includes: mood stabilizers, anxiolytics, antidepressants, and antipsychotics

^e^Psychiatric polypharmacy is defined as having overlapping coverage of ≥1 AP agent and ≥1 anxiolytic, antidepressant, or mood stabilizer for at least 60 consecutive days with no gaps larger than 7 days

^f^Reference: Elixhauser A, Steiner C, Kruzikas. D. HCUP Methods Series Report # 2004–1. ONLINE February 6, 2004. U.S. Agency for Healthcare Research and Quality. [Internet]. Comorbidity Software Documentation. Rockville, MD, USA; 2004 [cited 2013]. p. 12–5. Available from: http://www.hcup-us.ahrq.gov/reports/ComorbiditySoftwareDocumentationFinal.pdf

^g^Cardiovascular disease was defined using ICD-9-CM codes: 410.xx, 411.0×, 411.81, 411.89, 411.1×, 413.xx, 427.5×, 427.1×, 427.4×, 427.3×, 398.91, 428.xx, 414.0×, 414.1×, 414.8×, 414.9×, 429.3×, 402.9×, 430.xx, 431.xx, 432.xx, 433.xx, 434.xx, 435.xx, 436.xx, 437.xx, 427.89; hepatitis C was defined using the ICD-9 codes: 070.41, 070.44, 070.51, 070.54, 070.7×, V02.62

**Table 2:** Demographic and clinical characteristics evaluated during the 12-month baseline period in unweighted and IPT-weighted overall cohorts

	Overall cohort					
	Unweighted			IPT-weighted		
	PP1M (*N* = 2053)	OAA (*N* = 22,247)	*Std. Diff.* ^a^	PP1M (*N* = 11,612)	OAA (*N* = 12,688)	*Std. Diff.* ^a^
Age at index date (years), mean ± SD [median]	41.3 ± 12.6 [41.5]	43.9 ± 13.5 [45.0]	19.8%	42.9 ± 12.9 [43.6]	43.6 ± 13.4 [44.7]	5.1%
Female, %	39.2%	50.8%	23.6%	46.4%	49.6%	6.6%
Race, %						
White	49.5%	58.0%	17.0%	56.2%	57.1%	1.9%
Black	41.6%	33.1%	17.5%	34.2%	33.9%	0.6%
Hispanic	0.1%	0.2%	1.7%	0.3%	0.2%	1.7%
Other	6.1%	6.1%	0.1%	6.5%	6.1%	1.4%
Unknown	2.6%	2.6%	0.4%	2.8%	2.6%	1.5%
State, %						
Iowa	4.7%	6.6%	8.2%	7.2%	6.4%	3.2%
Kansas	10.2%	7.6%	9.1%	8.1%	7.9%	0.8%
Mississippi	12.3%	10.0%	7.4%	8.8%	10.2%	4.7%
Missouri	50.4%	50.4%	0.1%	52.4%	50.4%	3.9%
New Jersey	22.4%	25.4%	7.2%	23.5%	25.1%	3.7%
Region characteristics, %						
Urban	55.4%	56.2%	1.5%	55.4%	56.1%	1.4%
Suburban	26.7%	26.3%	1.0%	25.8%	26.2%	0.9%
Rural	17.9%	17.5%	0.9%	18.7%	17.7%	2.8%
Insurance eligibility, %						
Capitated or dual coverage	66.1%	66.7%	1.1%	67.1%	66.6%	1.2%
Capitated	40.4%	41.5%	2.1%	41.9%	41.5%	1.0%
Dual coverage	39.6%	39.9%	0.5%	40.4%	39.8%	1.3%
Year of index date, %						
2009	14.3%	12.4%	5.4%	9.9%	12.5%	8.3%
2010	38.6%	37.1%	3.2%	34.1%	37.2%	6.3%
2011	21.0%	23.8%	6.6%	21.7%	23.5%	4.3%
2012	13.1%	14.5%	4.1%	17.7%	14.4%	9.0%
2013	10.3%	9.5%	2.5%	13.0%	9.7%	10.4%
2014	2.7%	2.7%	0.4%	3.5%	2.7%	4.8%
Quan-CCI, mean ± SD [median]	0.7 ± 1.3 [0.0]	1.1 ± 1.7 [1.0]	26.7%	1.0 ± 1.6 [1.0]	1.1 ± 1.7 [1.0]	5.4%
Number of unique mental health diagnoses, mean ± SD [median]	7.2 ± 7.0 [5.0]	8.4 ± 8.5 [6.0]	15.0%	8.7 ± 8.1 [6.0]	8.3 ± 8.4 [6.0]	4.6%
Number of unique AP agents received, mean ± SD [median]	1.7 ± 1.1 [2.0]	1.0 ± 1.1 [1.0]	65.5%	1.3 ± 1.2 [1.0]	1.1 ± 1.1 [1.0]	17.7%
AP use, %	87.8%	61.8%	62.8%	67.9%	64.2%	8.0%
Typical oral and short-term injectable	24.2%	22.4%	4.2%	27.0%	22.8%	9.9%
Atypical oral and short-term injectable	65.7%	49.6%	33.0%	54.9%	51.0%	7.7%
Typical LAI	17.4%	6.0%	36.2%	8.5%	7.2%	4.9%
Atypical LAI	36.7%	5.3%	83.4%	9.1%	8.3%	2.8%
Proportion of days covered (PDC) by any AP agent, %						
PDC < 0.8^b^	64.3%	67.2%	6.2%	67.2%	66.9%	0.7%
PDC ≥ 0.8^b^	35.7%	32.8%	6.2%	32.8%	33.1%	0.7%
AP polypharmacy present^c^, %	22.9%	10.9%	32.6%	14.4%	12.1%	7.0%
Other psychiatric medication use, %	71.7%	76.8%	11.6%	75.2%	76.6%	3.1%
Antidepressants	50.1%	57.0%	13.9%	55.4%	56.4%	2.1%
Anxiolytics	40.1%	50.3%	20.6%	50.0%	49.4%	1.1%
Mood stabilizers	40.3%	41.0%	1.5%	42.9%	41.1%	3.6%
Number of unique psychiatric agents received^d^, mean ± SD [median]	3.6 ± 2.4 [3.0]	3.3 ± 2.6 [3.0]	11.3%	3.4 ± 2.7 [3.0]	3.3 ± 2.6 [3.0]	4.6%
Psychiatric polypharmacy present^e^, %	44.5%	37.2%	14.9%	39.6%	38.1%	3.0%
Specific comorbidities present^f^, %						
Cardiovascular disease^g^	11.2%	18.3%	20.1%	15.6%	17.6%	5.6%
Diabetes	20.5%	24.0%	8.6%	23.1%	23.7%	1.5%
Obesity	9.4%	10.4%	3.3%	12.9%	10.4%	7.9%
Drug abuse	21.5%	19.9%	4.0%	21.2%	20.1%	2.8%
Hepatitis C^g^	3.4%	4.7%	6.6%	4.2%	4.5%	1.6%
HIV/AIDS	1.3%	1.8%	4.3%	2.1%	1.8%	2.3%
Baseline monthly healthcare costs (2015 $US), mean ± SD [median]						
Total healthcare costs	$2050 ± $2848 [$1288]	$2230 ± $3883 [$1125]	5.3%	$2091 ± $3162 [$1098]	$2226 ± $3818 [$1154]	3.9%
Total pharmacy costs	$510 ± $762 [$107]	$363 ± $677 [$54]	20.4%	$420 ± $746 [$47]	$372 ± $678 [$57]	6.7%
Total medical costs	$1540 ± $2581 [$843]	$1867 ± $3727 [$783]	10.2%	$1671 ± $2855 [$777]	$1854 ± $3661 [$802]	5.6%
Baseline healthcare resource utilization, % with ≥1 visit/service						
Outpatient visits	91.1%	91.7%	2.2%	92.4%	91.7%	2.6%
Number of visits per month, mean ± SD [median]	1.06 ± 1.54 [0.67]	1.33 ± 2.25 [0.83]	13.6%	1.22 ± 2.04 [0.75]	1.31 ± 2.22 [0.75]	4.3%
Emergency room visits	48.4%	56.5%	16.2%	53.5%	56.0%	5.1%
Number of visits per month, mean ± SD [median]	0.16 ± 0.49 [0.00]	0.20 ± 0.56 [0.08]	7.4%	0.19 ± 0.51 [0.08]	0.20 ± 0.56 [0.08]	2.1%
Inpatient visits	46.7%	54.2%	15.2%	55.9%	53.9%	3.9%
Number of visits per month, mean ± SD [median]	0.11 ± 0.28 [0.00]	0.13 ± 0.26 [0.08]	7.7%	0.13 ± 0.25 [0.08]	0.13 ± 0.26 [0.08]	0.4%
Long-term care admissions	4.6%	8.3%	15.0%	6.9%	8.1%	4.7%
Number of admissions per month, mean ± SD [median]	0.01 ± 0.04 [0.00]	0.02 ± 0.10 [0.00]	12.6%	0.01 ± 0.05 [0.00]	0.02 ± 0.10 [0.00]	8.3%
Mental health institute admissions	62.6%	39.5%	47.4%	51.4%	41.3%	20.5%
Number of admissions per month, mean ± SD [median]	0.73 ± 1.18 [0.17]	0.36 ± 0.86 [0.00]	35.5%	0.45 ± 0.91 [0.08]	0.40 ± 0.90 [0.00]	5.5%
One-day mental health institute admissions	72.0%	53.2%	39.6%	61.1%	54.6%	13.1%
Number of admissions per month, mean ± SD [median]	1.28 ± 1.47 [0.75]	0.70 ± 1.16 [0.08]	43.7%	0.81 ± 1.18 [0.25]	0.76 ± 1.21 [0.08]	4.0%
Home care	33.9%	37.0%	6.4%	35.8%	36.9%	2.2%
Number of services per month, mean ± SD [median]	0.76 ± 2.73 [0.00]	1.10 ± 3.92 [0.00]	10.2%	0.85 ± 3.19 [0.00]	1.09 ± 3.88 [0.00]	6.9%
Other services	34.3%	33.5%	1.8%	32.2%	33.3%	2.3%
Number of services per month, mean ± SD [median]	0.12 ± 0.32 [0.00]	0.13 ± 0.41 [0.00]	3.8%	0.11 ± 0.29 [0.00]	0.13 ± 0.41 [0.00]	6.4%

*AP* antipsychotics, *CCI* Charlson comorbidity index, *IPT* inverse probability of treatment, *LAI* long-acting injectable therapy, *OAA* oral atypical antipsychotics, *PDC* proportion of days covered, *PP1M* once-monthly paliperidone palmitate

^a^Baseline characteristics with a standardized difference <10% were considered well balanced. For continuous variables, the standardized difference is calculated by dividing the absolute difference in means of the PP1M and the OAA cohorts by the pooled standard deviation of both groups. The pooled standard deviation is the square root of the average of the squared standard deviations. For categorical variables with 2 levels, the standardized difference is calculated using the following equation where P is the respective proportion of participants in each group: (P_PP1M_-P_OAA_)/√[p(1-p)], where p = (P_PP1M_ + P_OAA_)/2

^b^The denominator for the proportion of patients with a PDC <0.8 or a PDC ≥0.8 is the number of patients with a PDC >0

^c^AP polypharmacy is defined as having overlapping coverage of ≥2 unique AP agents for at least 60 consecutive days with no gaps larger than 7 days

^d^Includes: mood stabilizers, anxiolytics, antidepressants, and antipsychotics

^e^Psychiatric polypharmacy is defined as having overlapping coverage of ≥1 AP agent and ≥1 anxiolytic, antidepressant, or mood stabilizer for at least 60 consecutive days with no gaps larger than 7 days.

^f^Reference: Elixhauser A, Steiner C, Kruzikas. D. HCUP Methods Series Report # 2004–1. ONLINE February 6, 2004. U.S. Agency for Healthcare Research and Quality. [Internet]. Comorbidity Software Documentation. Rockville, MD, USA; 2004 [cited 2013]. p. 12–5. Available from: http://www.hcup-us.ahrq.gov/reports/ComorbiditySoftwareDocumentationFinal.pdf

^g^Cardiovascular disease was defined using ICD-9-CM codes: 410.xx, 411.0×, 411.81, 411.89, 411.1×, 413.xx, 427.5×, 427.1×, 427.4×, 427.3×, 398.91, 428.xx, 414.0×, 414.1×, 414.8×, 414.9×, 429.3×, 402.9×, 430.xx, 431.xx, 432.xx, 433.xx, 434.xx, 435.xx, 436.xx, 437.xx, 427.89; hepatitis C were defined using the ICD-9 codes: 070.41, 070.44, 070.51, 070.54, 070.7×, V02.62

Prior to weighting, recently diagnosed patients initiated on PP1M were older (mean age [SD]:22.3 [1.9] vs. 21.6 [2.0] years, standardized difference: 31.7%) relative to OAA patients. Recently diagnosed PP1M patients were less likely to be female (24.7% vs. 41.1%, standardized difference: 35.4%), more likely to have prior AP use (88.1% vs. 59.9%, standardized difference: 67.9%) and AP polypharmacy (19.8% vs. 8.8%, standardized difference: 32.0%), while patients treated with OAA had a higher baseline comorbidity index (mean CCI [SD]: 0.3 [0.6] vs. 0.4 [0.8], standardized difference: 11.5%; Table 1).

In the overall cohort and prior to weighting, patients initiated on PP1M were younger (mean age [SD]: 41.3 [12.6] vs. 43.9 [13.5] years, standardized difference: 19.8%) and less likely to be female (39.2% vs. 50.8%, standardized difference: 23.6%). Patients treated with PP1M were more likely to have prior AP use (87.8% vs. 61.8%, standardized difference: 62.8%) and AP polypharmacy (22.9% vs. 10.9%, standardized difference: 32.6%), while patients treated with OAA had a higher baseline comorbidity index (mean CCI [SD]: 1.1 [1.7] vs. 0.7 [1.3], standardized difference: 26.7%), prevalence of specific comorbidities (e.g., diabetes, cardiovascular disease), and baseline medical costs (mean monthly cost [SD]: $2230 [3883] vs. $2050 [2848], standardized difference: 5.3%; Table 2).

After IPT-weighting, the baseline characteristics were generally well balanced between PP1M and OAA cohorts for both the recently diagnosed cohort and the overall cohort.

### Treatment patterns

IPT-weighted treatment patterns assessed during the observation period are described in Table 3. In recently diagnosed patients, 56.3% of PP1M patients used another AP during follow-up as compared to 67.9% of OAA patients (*P* < 0.001), with a lower proportion of PP1M patients using other concomitant psychiatric medications as compared to OAA patients (69.0% vs. 87.2%, *P* < 0.001). Furthermore, the proportion of PP1M patients with AP polypharmacy (16.6% vs. 26.3%, *P* < 0.001) and with psychiatric polypharmacy (45.2% vs. 62.4%, *P* < 0.001) were both significantly lower than that of the OAA cohort. In the recently diagnosed cohort, PP1M patients were also more likely to be adherent to the index medication (PDC ≥80%: 28.8% vs. 21.1%, *P* < 0.001) and persistent on the index medication (no gap ≥60 days: 38.7% vs. 27.6%; *P* < 0.001) as compared to OAA patients.

**Table 3:** Treatment patterns assessed during the 12-month follow-up using IPT-weighted cohorts

	IPT-weighted recently diagnosed cohort			IPT-weighted overall cohort		
	PP1M	OAA	*P*-value^a^	PP1M	OAA	*P*-value^a^
	(*N* = 1107)	(*N* = 1288)		(*N* = 11,612)	(*N* = 12,688)	
Duration of continuous exposure to index agent (days)^b^, mean ± SD [median]	217.7 ± 128.4 [225.0]	189.4 ± 123.2 [152.0]	0.0017*	215.8 ± 128.8 [193.0]	194.4 ± 123.1 [160.0]	<.0001*
Number of dispensings of index agent, mean ± SD [median]	7.7 ± 4.4 [8.0]	7.9 ± 6.4 [6.0]	0.4888	7.8 ± 4.4 [7.0]	8.0 ± 5.8 [7.0]	0.1489
Adherence (PDC ≥ 80%) on index agent, %	28.8%	21.1%	<.0001*	26.7%	22.7%	<.0001*
PDC on index agent, mean ± SD [median]	0.53 ± 0.30 [0.55]	0.47 ± 0.30 [0.41]	0.0062*	0.53 ± 0.30 [0.52]	0.49 ± 0.30 [0.44]	<.0001*
Adherence (PDC ≥ 80%) on any AP agent, %	43.2%	40.2%	0.1420	39.3%	38.4%	0.1534
PDC on any AP agent, mean ± SD [median]	0.65 ± 0.28 [0.71]	0.64 ± 0.29 [0.70]	0.7028	0.63 ± 0.29 [0.69]	0.64 ± 0.28 [0.67]	0.3509
Persistence on index agent, %						
No gap ≥30 days	21.8%	18.0%	0.0203*	21.9%	19.1%	<.0001*
No gap ≥60 days	38.7%	27.6%	<.0001*	37.8%	29.0%	<.0001*
No gap ≥90 days	46.9%	35.1%	<.0001*	43.8%	36.1%	<.0001*
Persistence on any AP agent, %						
No gap ≥30 days	31.2%	35.0%	0.0548	32.9%	33.0%	0.8544
No gap ≥60 days	53.2%	50.3%	0.1518	48.9%	47.1%	0.0046*
No gap ≥90 days	64.8%	59.9%	0.0132*	56.5%	56.0%	0.3737
Psychiatric medication use during follow-up (excluding index agent), %	73.4%	93.5%	<.0001*	82.1%	94.8%	<.0001*
AP use	56.3%	67.9%	<.0001*	59.1%	64.8%	<.0001*
Typical oral and short-term injectable	21.7%	26.1%	0.0128*	22.3%	24.9%	<.0001*
Atypical oral and short-term injectable	52.9%	52.6%	0.8815	49.5%	48.4%	0.0892
Typical LAI	3.0%	5.9%	0.0010*	4.6%	6.9%	<.0001*
Atypical LAI	4.6%	14.0%	<.0001*	2.9%	11.5%	<.0001*
Other psychiatric medication use	69.0%	87.2%	<.0001*	75.2%	89.5%	<.0001*
Anxiolytics	31.9%	50.7%	<.0001*	47.6%	58.4%	<.0001*
Mood stabilizers	40.0%	56.4%	<.0001*	40.6%	52.5%	<.0001*
Antidepressants	49.6%	66.8%	<.0001*	54.5%	70.1%	<.0001*
AP polypharmacy present^c^, %	16.6%	26.3%	<.0001*	23.6%	29.2%	<.0001*
Psychiatric polypharmacy present^d^, %	45.2%	62.4%	<.0001*	49.7%	67.7%	<.0001*

*AP* antipsychotics, *IPT* inverse probability of treatment, *LAI* long-acting injectable therapy, *OAA* oral atypical antipsychotics, *PDC* proportion of days covered, *PP1M* once-monthly paliperidone palmitate

^a^Calculated using the Pearson chi-square test (categorical variables) and Student’s t-test (continuous variables)

^b^Continuous exposure with the agent (PP1M or specific OAA) that was used to define the index date. Continuous therapy was defined as having a gap of no more than 90 days between two claims for the index agent

^c^AP polypharmacy is defined as having overlapping coverage of ≥2 unique AP agents for at least 60 consecutive days with no gaps larger than 7 days.

^d^Psychiatric polypharmacy is defined as having overlapping coverage of ≥1 AP agent and ≥1 anxiolytic, antidepressant, or mood stabilizer for at least 60 consecutive days with no gaps larger than 7 days

*indicates that *p*-value <0.05

In the overall cohort, differences between PP1M and OAA patients generally appeared smaller than those observed for the recently diagnosed cohort. Overall, PP1M was associated with having a longer continuous exposure to the index treatment (mean days [SD]: 215.8 [128.8] vs. 194.4 [123.1], *P* < 0.001), and with less use of other APs (59.1% vs. 64.8%, *P* < 0.001) or other psychiatric medications (i.e., anxiolytics, antidepressants, or mood stabilizers; 75.2% vs. 89.5%, *P* < 0.001) in addition to the index treatment. Furthermore, PP1M patients were less likely to have AP polypharmacy (23.6% vs. 29.2%, *P* < 0.001) or psychiatric polypharmacy (49.7% vs. 67.7%, *P* < 0.001) compared to OAA patients. Moreover, in the overall cohort, PP1M patients were more likely to have a PDC ≥80% for the index medication (26.7% vs. 22.7%, *P* < 0.001) and no gap ≥60 days on the index medication (37.8% vs. 29.0%; *P* < 0.001) relative to OAA patients at 12 months of follow-up.

### Healthcare costs

**Fig. 2:**
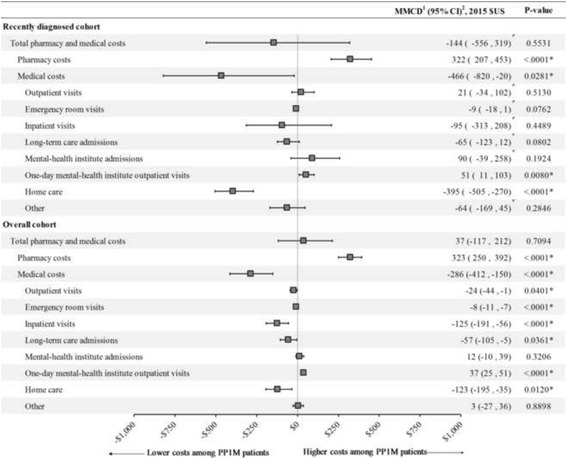
Mean monthly cost differences for PP1M and OAA IPT-weighted cohorts during the 12-month follow-up. CI = confidence interval; IPT = inverse probability of treatment; OAA = oral atypical antipsychotics; PP1M = once-monthly paliperidone palmitate; MMCD = mean monthly cost difference. * indicates *p*-value <0.05. ^1^All linear models included a binary indicator for the treatment received at the index date. ^2^Based on bootstrap results (B = 499)

Figure 2 describes the mean monthly cost differences between IPT-weighted PP1M and OAA cohorts during the observation period for recently diagnosed and overall cohorts, respectively. In the recently diagnosed cohort, PP1M was associated with lower medical costs (MMCD = $-466 [−820, −20], *P* = 0.028), which were driven mainly by lower home care costs (MMCD = $-395 [−505, −270], *P* < 0.001). The medical cost savings observed with PP1M in the recently diagnosed cohort outweighed the higher pharmacy costs, resulting in similar total healthcare costs relative to OAA (MMCD = $-144 [−556, 319], *P* = 0.553).

In the overall cohort, PP1M patients had significantly lower medical costs compared to OAAs (MMCD = $-286 [−412, −150], *P* < 0.001) offsetting most of the higher pharmacy costs (MMCD = $323 [250, 392], *P* < 0.001), resulting in similar total healthcare costs for both treatment groups (MMCD = $37 [−117, 212]; *P* = 0.709). Medical cost savings associated with PP1M in the overall cohort appeared to be mainly driven by lower inpatient costs (MMCD = $-125 [−191, −56]; *P* < 0.001) and lower home care costs (MMCD = $-123 [−195, −35]; *P* = 0.012).

In a sensitivity analysis applying the 23.1% minimum mandatory discount to branded pharmaceutical products, among recently diagnosed patients, total pharmacy costs remained higher for patients initiated on PP1M (MMCD = $233 [139, 340]; *p* < 0.001) and total healthcare costs were not significant different between PP1M and OAA cohorts (MCCD = $-233 [−635, 228]; *p* = 0.317). Similarly in the overall cohort, total pharmacy costs remained higher for patients initiated on PP1M (MMCD = $240 [180, 304]; *p* < 0.001) and total healthcare costs were similar between cohorts (MMCD = $-46 [−197, 123]; *p* = 0.533).

### Healthcare resource utilization

Among recently diagnosed patients, the rate of home care services was significantly lower with PP1M (RR [95% CI] = 0.43 [0.18, 0.77]; *P* = 0.008) while the rates of mental health institute admissions (RR [95% CI] = 1.44 [1.01, 1.98]; *P* = 0.044) and 1-day mental health institute admissions (RR [95% CI] = 1.31 [1.04, 1.57]; *P* = 0.028) were significantly higher compared to OAA. Cumulative lengths of stay for all-cause inpatient visits (RR [95% CI] = 0.85 [0.58, 1.19]; *P* = 0.353) and long-term care admissions (RR [95% CI] = 0.40 [0.07, 1.12]; *P* = 0.060) also appeared shorter among PP1M relative to OAA patients in the recently diagnosed cohort, although these findings were not statistically significant. PP1M was also associated with significantly lower rates of long-term care admissions (RR [95% CI] = 0.17 [0.04, 0.35]; *P* < 0.001) as compared to OAAs.

**Fig. 3:**
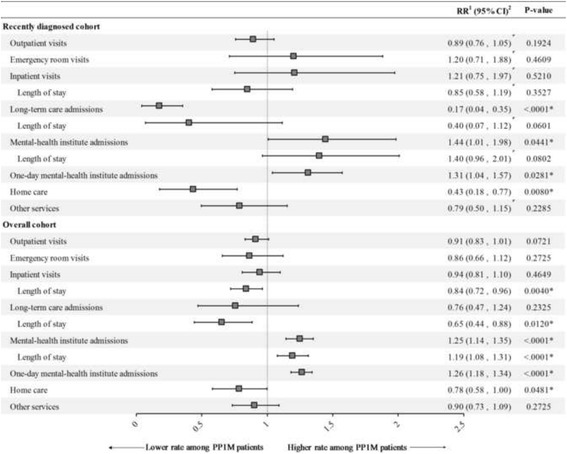
Healthcare resource utilization for PP1M and OAA IPT-weighted cohorts during the 12-month follow-up. CI = confidence interval; IPT = inverse probability of treatment; OAA = oral atypical antipsychotics; PP1M = once-monthly paliperidone palmitate; MMCD = mean monthly cost difference. * indicates *p*-value <0.05. ^1^All Poisson models included a binary indicator for the treatment received at the index date. ^2^Based on bootstrap results (B = 499)

Among the overall cohort, treatment with PP1M was associated with having 16% fewer all-cause inpatient days (RR [95% CI] = 0.84 [0.72, 0.96]; *P* = 0.004), 35% fewer long-term care days (RR [95% CI] = 0.65 [0.44, 0.88]; *P* = 0.012), and a 22% lower rate of home care visits (RR [95% CI] = 0.78 [0.58, 1.00]; *P* = 0.048) relative to treatment with OAA (Fig. 3). However, the rates of mental health institute admissions (RR [95% CI] = 1.25 [1.14, 1.35]; *P* < 0.001), 1-day mental health institute admissions (RR [95% CI] = 1.26 [1.18, 1.34]; *P* < 0.001), and cumulative length of stay for mental health institute admissions (RR [95% CI] = 1.19 [1.08, 1.31]; *P* < 0.001) were all significantly higher in PP1M patients versus OAA patients. For all other categories of resource utilization (i.e., outpatient visits, emergency room visits, inpatient visits, long-term care admissions, and other services), the point estimates suggested a lower rate of visits/services among PP1M patients as compared to OAA patients; however, these findings were not significant (*P* > 0.05).

## Discussion

This study aimed to provide real world evidence on the impact of PP1M relative to OAA treatment for patients recently diagnosed with schizophrenia and within the overall cohort of patients with schizophrenia. Results for recently diagnosed patients (defined as those aged 18–25) indicate that PP1M is associated with increased rates of adherence and persistence after 12 months of observation; lower AP and psychiatric polypharmacy; reduced frequency of home care services; and lower medical costs relative to OAA in the treatment of schizophrenia. These outcomes fully offset the increased pharmacy costs associated with PP1M, resulting in similar total healthcare costs as OAA therapy within the 12-month observation period. PP1M was also associated with increased frequency of mental institute and 1-day mental institute admissions among recently diagnosed patients.

Among the overall cohort of patients diagnosed with schizophrenia, PP1M treatment also appeared to be associated with lower adherence and persistence and lower medical costs, compared to OAA. While total healthcare costs were similar for PP1M and OAA treated patients in the overall population and in the recently diagnosed cohort, results trended toward total healthcare cost savings for the recently diagnosed cohort and suggested a larger magnitude of medical cost savings than that observed in the overall population. While Medicaid claims data did not account for rebates provided by manufacturers, a sensitivity analysis was performed that applied a 23.1% rebate assumption (the mandatory minimum discount provided to Medicaid) [19] on branded pharmaceutical products. This sensitivity analysis found that the excess pharmacy costs observed in the PP1M cohort versus OAA were approximately $80–$90 lower per patient per month, suggesting that our study likely overestimated pharmacy costs particularly for the PP1M cohort. Notably, the conclusions of the study were not sensitive to this change. Given that the 23.1% used represents the minimum discount provided, these sensitivity analyses may still overestimate pharmacy costs and underestimate the net pharmacy cost difference between PP1M and OAA.

For the overall population, the findings of the current study on healthcare costs and resource utilization are consistent with those of previous studies using claims or hospital databases [11, 25–28]. Xiao et al. showed that PP1M was associated with comparable overall cost to OAAs, but with significantly lower medical costs, particularly driven by reductions in inpatient visits in Medicaid beneficiaries [27]. In a matched cohort of Veterans Affairs (VA) patients with schizophrenia, Baser et al. found that treatment with PP1M was associated with lower inpatient costs, higher pharmacy costs, and non-different total healthcare costs, as well as lower inpatient admission rates [25]. Morrato et al., in an observational comparative effectiveness study among Medicaid patients from the state of Missouri, showed that patients starting treatment with PP1M versus OAAs were less likely to have ER visits and hospitalizations although the difference in hospitalizations did not achieve statistical significance [26]. A retrospective cohort study using hospital discharge and billing records also showed that PP1M versus OAA treatment in an inpatient setting was associated with lower risks of all-cause re-hospitalization and ER use and lower institution costs [11]. Another recent study by Pesa et al. also examined resource use and costs in patients iniated on PP1M versus oral APs using U.S. commercial claims data [28]. Findings by Pesa et al. were similar to those in the overall cohort of the present study, including higher pharmacy costs but lower medical costs over a 12-month follow-up period [28].

Despite literature suggesting that recently diagnosed patients with schizophrenia can be treated safely and efficaciously with long-acting injectable therapies including PP1M, they are not commonly used in this population [9, 16, 29, 30]. Reasons for this may be multifactorial, but may be due in part to prescribing psychiatrists’ concerns over tolerability and patient acceptance, and patient lack of awareness of LAI as a treatment option [31, 32]. However, patients early in their course of schizophrenia may present an opportunity for intervention and control of symptoms before recurrent relapse and associated challenges have a chance to act as barriers to effective treatment [29, 33]. In particular, recently diagnosed patients are important candidates for LAI due to their high risk for non-adherence issues, which may especially affect this population due to poor insight into illness, poor attitudes toward medication, and higher rates of problematic substance use [8, 9]. Indeed, among patients with first-episode psychosis, rates of AP non-adherence may exceed 50% [33], with approximately 30% of patients discontinuing therapy within 9 months [9]. Consistent with this information, LAI should be considered earlier in the treatment of schizophrenia [34, 35], with some clinical guidelines recommending them in early schizophrenia [29] or as first-line treatment in schizophrenia patients (in the case of SG-LAI) [36].

The present study builds on previous results from an open-label, randomized study of patients with schizophrenia who had a history of criminal justice involvement [18]. Alphs et al. observed that PP1M may be associated with a greater impact on treatment discontinuation versus oral APs among patients with recent-onset illness compared to those with chronic illness [18]. While there was a trend toward a greater impact of PP1M among patients with recent-onset illness, the findings were not statistically significant in this cohort. Another previous study also demonstrated that PP1M is associated with longer time-to-relapse (85% of patients relapse-free at 469 days versus 249 days) and a 29.4% reduction in the relative risk of relapse over 24-months compared to oral APs among patients diagnosed within the past 1–5 years [17].

Nonetheless, to the best knowledge of the authors, this study is the first to evaluate the impact of PP1M relative to OAA on adherence, healthcare costs, and healthcare resource utilization in Medicaid-covered patients recently diagnosed with schizophrenia in a real-world setting. The population of patients aged 18–25 used in the present study was selected in order to best approximate recently diagnosed patients, given that the peak age of schizophrenia onset is early-to-mid 20s [3]. Based on indirectly comparing the magnitude of benefits observed for recently diagnosed patients to that of the overall cohort, results appear to support the hypothesis that recently diagnosed patients experience a greater benefit from PP1M than from OAAs; however, further investigations are warranted to better understand the importance of this finding.

In this study, we found evidence that patients initiated on PP1M used fewer additional psychiatric medications (i.e., other antipsychotics, antidepressants, anxiolytics, and mood stabilizers) and were less likely to have antipsychotic polypharmacy as compared to patients initiated on OAA, which could contribute to reducing prescription-related healthcare resource utilization; to reducing the potential negative impacts of a high level of polypharmacy; [37] and improving adherence. Additional studies may be warranted to further explore any impacts of PP1M on AP polypharmacy and its subsequent outcomes. While it was not examined in the scope of this investigation, indirect impacts of PP1M on non-schizophrenia-related costs and resource utilization are another possible explanation for the observed association between PP1M and medical cost savings. Previous investigations have highlighted the high comorbidity burden and comorbidity-related costs among schizophrenia patients [38], leading to the hypothesis that improved management of schizophrenia could improve the management of other health conditions.

The current study was subject to certain limitations that warrant mention. First, the Medicaid data used in this study came from only five states during a limited study period and may not be representative of the United States, of other states, or of non-Medicaid patients. As required by federal law, Medicaid coverage is available for certain groups including low income families and blind and disabled persons. However, specific eligibility criteria and coverage details may differ across states. For example, Medicaid expansion, an opportunity to expand coverage to all low-income individuals under 65, was not adopted by three out of the five states included in this study (KS, MO, MS) [39]. It is also worth mentioning that these data spanned for approximately 5 years following the approval of PP1M; as new data become available, it will be important to compare these findings and to consider the possible impacts of learning and rollout following the initial drug approval. Second, the data, coming from claims, were subject to billing inaccuracies and missing information (e.g., data entry errors, diagnoses and procedures coded inaccurately), although such issues are expected to affect both cohorts similarly. Third, claims-based adherence measures such as PDC or persistence do not account for whether the drugs dispensed were actually taken as prescribed. This may overestimate patient adherence, especially the OAA cohort, for whom we assumed that they take their medication correctly (e.g., one pill per day), whereas for PP1M, the effect of one injection stays in the patient’s body for the length of the prescription without any other action required by the patient. Given that we imposed at least two prescriptions for inclusion in the study, it is also possible that patients with very low adherence/persistence may have been excluded by design. Fourth, as cohorts were determined at the index date in this study (“intent-to-treat” approach), interpretation of the estimates may become difficult if a large proportion of participants crossed over between the treatment arms or concomitantly used both treatments. In this study after weighting, 48.6% of PP1M patients used an OAA during the 12 months of observation (includes OAAs that may have been used prior to PP1M initiation) and 4.9% and 7.6% of OAA patients used PP1M or another LAI during that same period, respectively. The higher add-on/switch rates to an OAA among the PP1M cohort likely makes these results conservative as the benefits observed in the PP1M cohort may have been muted. Fifth, while age as a proxy for being recently diagnosed was informed by the mean age of onset of schizophrenia (i.e., typically in the early-to-mid 20s for men and in the late 20s for women) [3], this definition may lack precision. One reason for this may be that some patients experience long delays between the appearance of symptoms and initiation of treatment [7], preventing the identification of patients at illness onset. Young age was used to maximize the specificity of the definition of recently diagnosed (i.e., since very few patients are expected to experience disease onset before this age) but at the possible risk of underrepresenting patients with late onset disease and women who tend to experience later onset. Given that age defined the recently diagnosed cohort in this study and that this cohort comprised a relatively small proportion of the overall cohort of patients, further investigation is needed to understand whether these findings are generalizable to recently diagnosed patients of other ages or who have been identified using other criteria (e.g., using information about the date of first diagnosis) Finally, as with all retrospective administrative claims data, the study results may be subject to residual confounding due to unmeasured confounders. Nonetheless, even though health insurance claims data present such shortcomings, they remain a valuable source of information because they contain a fairly valid and large sample of patients’ characteristics and outcomes in a real-world setting.

## Conclusions

A higher proportion of PP1M patients were adherent to therapy after 12 months compared to OAA patients, both for the overall and recently diagnosed cohorts. Overall, Medicaid enrollees with schizophrenia treated with PP1M demonstrated significantly lower medical costs, which fully offset higher pharmacy costs during 12-months follow-up resulting in similar total healthcare costs relative to patients treated with OAAs. Medical cost savings were mainly driven by fewer inpatient days and fewer home care visits for PP1M versus OAA patients. Results among recently diagnosed patients (aged 18–25 years) treated with PP1M appeared similar to the overall population, but suggested a greater magnitude of medical cost savings versus OAA as compared to that of the overall PP1M-treated population, highlighting the potential economic impact of using PP1M in adults recently diagnosed with schizophrenia. One potential explanation for the greater cost savings observed for recently diagnosed PP1M-treated patients is the larger observed difference in index medication adherence and persistence for PP1M- versus OAA-treated patients; further investigation is needed to confirm this association.

## Abbreviations

AP: Antipsychotic
CI: Confidence interval
IPT: Inverse probability of treatment
LAI: Long-acting injectable therapy
MMCD: Mean monthly cost difference
OAA: Oral atypical antipsychotics
PP1M: Once-monthly paliperidone palmitate.
RR: Rate ratio
